# Prolactin Expression in the Cochlea of Aged BALB/c Mice Is Gender Biased and Correlates to Loss of Bone Mineral Density and Hearing Loss

**DOI:** 10.1371/journal.pone.0063952

**Published:** 2013-05-07

**Authors:** Robert J. Marano, Jennifer Tickner, Sharon L. Redmond

**Affiliations:** 1 Ear Science Institute Australia, Subiaco, Western Australia, Australia; 2 Ear Sciences Centre, School of Surgery, The University of Western Australia, Nedlands, Western Australia, Australia; 3 School of Pathology and Laboratory Medicine, QEII Medical Centre, The University of Western Australia, Nedlands, Western Australia, Australia; University of South Florida, United States of America

## Abstract

Prolactin is a versatile hormone with over 300 known functions and predominantly expressed in the pituitary. However, its expression has additionally been found in a number of extrapituitary organs. Recently, we described the expression of prolactin in the inner ear of mice, where it was correlated to age. Previous research has shown prolactin to be linked to abnormal bone metabolism and hearing loss due to changes in morphology of the bony otic capsule. Here we further investigated the relationship between prolactin, hearing loss and cochlea bone metabolism. BALB/c mice were tested for hearing using ABR at 6 and 12 months of age. Bone mineral density of the cochlea was evaluated using microCT scanning. Prolactin expression was calculated using quantitative real time PCR. Expression of the key regulators of bone metabolism, osteoprotegerin and receptor activator of nuclear factor-kappaB ligand were also determined. We found that prolactin expression was exclusive to the female mice. This also correlated to a greater threshold shift in hearing for the females between 6 and 12 months of age. Analyses of the cochlea also show that the bone mineral density was lower in females compared to males. However, no gender differences in expression of osteoprotegerin or receptor activator of nuclear factor-kappaB ligand could be found. Further analysis of cochlea histological sections revealed larger ostocyte lacunae in the females. These results provide a possible mechanism for an age related hearing loss sub-type that is associated with gender and provides clues as to how this gender bias in hearing loss develops. In addition, it has the potential to lead to treatment for this specific type of hearing loss.

## Introduction

Prolactin (*PRL*) is a helix bundle peptide hormone predominantly expressed by lactotrophic cells located in the anterior pituitary gland. Since its early discovery associated with promoting lactation in mammals or “crop milk” in birds [1], it is now known to possess over 300 biological activities (more than all the other pituitary hormones combined) and has been the subject of numerous detailed reviews [2]–[7]. In addition to the pituitary, localised *PRL* expression has been found in a number of other extrapituitary sites including the brain [8]–[11], reproductive organs, tissue associated with the immune system (lymphocytes, thymus and spleen) [7] and adipocytes [5].

One of the activities of *PRL* is a direct inhibitory effect on osteoblast function [12]. In addition, *PRL* enhances bone resorption by decreasing the expression ratio between osteoprotegerin (OPG) and receptor activator of nuclear factor-kappaB ligand (RANKL) in osteoblasts derived from humans, by both decreasing OPG and increasing RANKL [13]. Further to this, it has been shown that induced hyperprolactinemia in a guinea pig model leads to bone dismorphology of the otic capsule with an associated hearing loss [14].

Recently we conducted a microarray study comparing the relative expression of genes within the cochlea of mice aged between 4 weeks (young) and 45 weeks (old) [15]. Of the 117 genes that displayed differential expression between the young and old mice, *PRL* showed the highest levels of upregulation of 108 fold. However, during validation using qRT-PCR, it was found that *PRL* expression was not detectable in the young mice, but was readily detectable in the old mice. This result was unique in that *PRL* expression in the cochlea had not previously been described in the literature, and the expression was associated with age. In addition to *PRL*, the closely related hormone Growth Hormone (*Gh*) was also highly upregulated, and the potent *PRL* inhibitor calcitonin-a (*CalcA*) was downregulated.

Subsequent histological sectioning of the contralateral cochlea from the same mice, followed by immunostaining with antibodies specific to *PRL*, revealed spiral ganglion cells and marginal cells of the stria vascularis as the sole sites of expression. Further immunostaining using *PRL* receptor (*PRLR*) specific antibodies revealed a wide variety of cell types expressing *PRLR*, including osteocytes and bone lining cells (BLC). This provided an indication that *PRL* expression was potentially linked to abnormal bone metabolism or degeneration of other structures within the cochlea leading to an age-related hearing loss (ARHL) pathology.

We hypothesized that localised *PRL* expression in the cochlea leads to bone and/or tissue degeneration resulting in hearing loss. Therefore, in this study we have investigated for the presence of *PRL* and its association to hearing loss in a cohort of BALB/c mice. In addition, we investigated if changes in *Gh* and *CalcA* expression were linked to *PRL* expression as previously found. We also examined the bone mineral density (BMD) of the cochlea to determine if there was a correlation between *PRL* expression and bone dismorphology. Furthermore, the OPG:RANKL ratio was also calculated for each of the mice.

## Materials and Methods

### Auditory Brainstem Response Testing

Animal experiments were considered and approved by the Walter and Eliza Hall Institute animal ethics committee (project 2011.016) and performed in accordance with the Australian Code of Practice for the Care and Use of Animals for Scientific Purposes (7^th^ ed, 2004). The hearing of BALB/c mice (n = 9; 5 female and 4 male) were assessed with an Auditory Brainstem Response (ABR) test using a Tucker Davis Technologies (TDT) Evoked Potentials Workstation (Tucker Davis Technologies, Alachua, FL, USA) under conditions similar to that reported by Carpinelli *et al*. 2011 [16]. Briefly, the mice were anaesthetized and presented with computer-generated clicks (0–50 KHz spectrum) and pure-tone stimuli of 4, 8, 16 and 32 kHz at maximum intensities of 100 dB peak equivalent SPL from a TDT free-field magnetic speaker (FF1) with a Free Field Frequency response of 108 dB SPL at 10 cm from 1 KHz to 50 KHz using +/−4 V input. The speaker was placed 10 cm from the nose tip of the mouse. Speaker calibration was performed using an ACO model 7016 microphone (ACO Pacific, Inc. Belmont, CA, USA) also placed 10 cm from the speaker. Clicks used had a 100 µsec duration and 100 KHz sampling rate with a null at 10, 20, 30, 40 and 50 KHz. ABRs were averaged from 512 recordings and thresholds were defined as the lowest intensity stimulus that reproducibly elicited a Wave III ABR using a visual detection criterion. Results of the ABR were analyzed using a Wilcoxon Rank Sum/Mann-Whitney U-Test and Repeated Measures ANOVA. Differences were considered significant at p<0.05.

### RNA Extraction

RNA was extracted from the mice cochlea as previously described [15] using the AllPrep kit (QIAGEN Pty Ltd – Australia, Doncaster, VIC, Australia) as per the manufactures instructions. Briefly, under a dissecting microscope, the cochlea (including the bone) was separated from the vestibular organs and placed into a 1.5 ml microfuge tube (Thermo, Melbourne, VIC, Australia) containing extraction buffer and homogenized using a polypropylene, pellet pestle (Sigma-Aldrich, St Louis, MO, USA). The homogenized mixture was then centrifuged at full speed for five minutes (Eppendorf Centrifuge 5424, Eppendorf South Pacific Pty. Ltd., North Ryde, NSW, Australia) and the supernatant removed and used for the remainder of the extraction protocol. RNA was eluted in 40 µl of RNase free H_2_O. The concentration of RNA was measured using a NanoPhotometer™ (IMPLEN, München, Germany).

### cDNA Synthesis

Synthesis of first strand cDNA was performed using the SuperScript™ III Reverse Transcriptase (RT) kit (Invitrogen, Mulgrave, Australia) as per the manufactures instructions. Samples of the extracted RNA were used as the template in a final reaction volume of 20 µl with an incubation period of 60 minutes at 55°C. Samples were purified after first strand synthesis using Centri–Spin™-40 columns (Princeton Separations Inc., USA). The cDNA concentration was measured using the NanoPhotometer™ (IMPLEN), and adjusted to 1 ng/µl and stored at −20°C.

### Quantitative Real Time PCR (qRT-PCR)

For all experiments, primers were designed using Primer – BLAST (National Library of Medicine) and consist of the following: (sequences given in 5′–3′) PRL Forward GCC CCA CTT CTT CCC TGG CT; Reverse GGG CAA TTT GGC ACC TCA GGA (411 bp); Gh Forward TGC CCA GGC TGC TTT CTG CT, Reverse ACC CGC AGG TAG GTC TCC GC (393 bp); CalcA Forward AGG CGC TGG GAG GCA CAG GAG CCA, Reverse AGG CCT GAA GGT CCC TGC GGC GG (508 bp); OPG Forward AGC ACT GAC CCA GCG GCT GCC TCC T, Reverse GGA GCC CGG GGG ACA GCT CCG GT (429 bp); RANKL Forward AGC GGC CCC GGC GTC CCA CA, Reverse GGC TGG GCC TCA GGC TTG CC TCG C (431 bp) and the housekeeping gene GAPDH Forward CCG CCC CTT CTG CCG ATG CCC CC, Reverse GCA GCC CCA CGG CCA TCA CGC C (244 bp). Reaction components per tube were as follows: 10 µl of iQ™ iSYBR Green® supermix (Bio-Rad Laboratories, UK), 1 pmol each forward and reverse primer and 5 ng of template cDNA with the final volume adjusted to 20 µl with ddH_2_O. PCR was performed using an iCycler™ MyiQ™ (Bio-Rad) with the cycling conditions consisting of an initial, two-step melt and extension of 95°C for two minutes and 68°C for two minutes respectively. This was followed by 40 cycles of 95°C–15 seconds and 68°C–30 seconds. Technical repeats were performed for each sample and the average C_T_ values for each gene and gender were used to calculate the relative gene expression for *PRL*, *Gh* and *CalcA* using the Livak Method (2^−ΔCT^) [17]. The OPG:RANKL ratio was calculated using the 2^−ΔΔCT^ method. Samples of the amplicons were also run on a 1.5% agarose gel at 90 V for 45 minutes, visualized on a UV transilluminator and photographed with a digital camera. Statistical analysis of expression levels and ratio's between males and females were analyzed using a Students T-test. Differences were considered significant at p<0.05.

### Histology and Staining

Mouse cochlea were fixed in 4% paraformaldehyde, pH 8.0 and decalcified at 40°C in a 14% EDTA w/v solution for 48 hours on a rocker shaker (100 rpm) (Ratek, Boronia, VIC, Australia). Cochlea tissue were then routinely processed overnight in a Leica automated tissue processor (model TP1020, Leica, Australia), paraffin embedded and sectioned at 4 µm. Sections were deparaffinised through xylene, graded ethanol and rinsed in tap water before commencement of staining.

#### Tartrate-resistant acid phosphatase (TRAcP) staining

Sections were immersed in TRAcP stain (pH 5.0) solution at 37°C and monitored every 30 minutes for two hours. Sections were then lightly counterstained in Gill's haematoxylin, washed well and mounted in an aqueous mounting medium. Positive control samples were stained in parallel and consisted of mouse femur known to contain active osteoclasts.

#### Haematoxylin and Eosin (H&E)

Sections were deparaffinised through xylene, graded ethanols, rinsed in tap water and placed in Gills haematoxylin for five minutes. Slides were washed well in tap water followed by blueing in saturated lithium carbonate for two minutes. Slides were then washed well in running tap water followed by one minute in 1% alcoholic eosin, dehydration in graded ethanols and xylene. Sections were permanently coverslipped in DePeX mounting media (Sigma-Aldrich, Sydney, Australia). Stained sections were digitally scanned using an Aperio ScanScope XT automated slide scanner (Aperio Technologies Inc., Vista, CA) with a 2× doubler inserted.

### Analysis of Bone

#### Bone Mineral Density (BMD) Measurement

Water controls, cochlea samples, and phantoms were scanned at 6.1 µm resolutions in a Skyscan 1174 microCT instrument (Skyscan, Aartselaar, Belgium). Source voltage was 50 kV, current of 800 µA, rotation step 0.4°, with a 0.5 mm aluminium filter. Scans were reconstructed using NRecon software (cone beam reconstruction algorithm, Skyscan) with a constant global threshold. Reconstructed samples were then imported into CTan software (Skyscan) for 3D analysis. BMD was measured by CTan, which was calibrated using phantoms of known BMD (0.25 g/cm^3^ and 0.75 g/cm^3^). All water controls, phantoms, and samples were scanned, reconstructed, and analysed under the same conditions, and samples were blinded prior to scanning. Results of BMD measurements were statistically compared between female and male mice using a Students T-test. Results were considered significant at p<0.05.

#### Area of Lacunae

Images from the scanned slides of H&E stained sections were visualised using Aperio ImageScope™ software (v11.2.0.780, Aperio Technologies). A combined total of 100 osteocyte lacuna were randomly chosen from the sections of both female and male mice. The areas of the lacunae were calculated by measuring the diameter of circular lacuna and applying the formula area  =  π × radius^2^. The area of elliptical lacunae was calculated by measuring the length of the major (Maj) and minor axis (Min) and applying the formulae area  =  (0.5× Maj) × (0.5× Min) × π. Data were analysed using a Students T-test and were considered significant at p<0.05.

## Results

### Gene Expression

**Figure 1:**
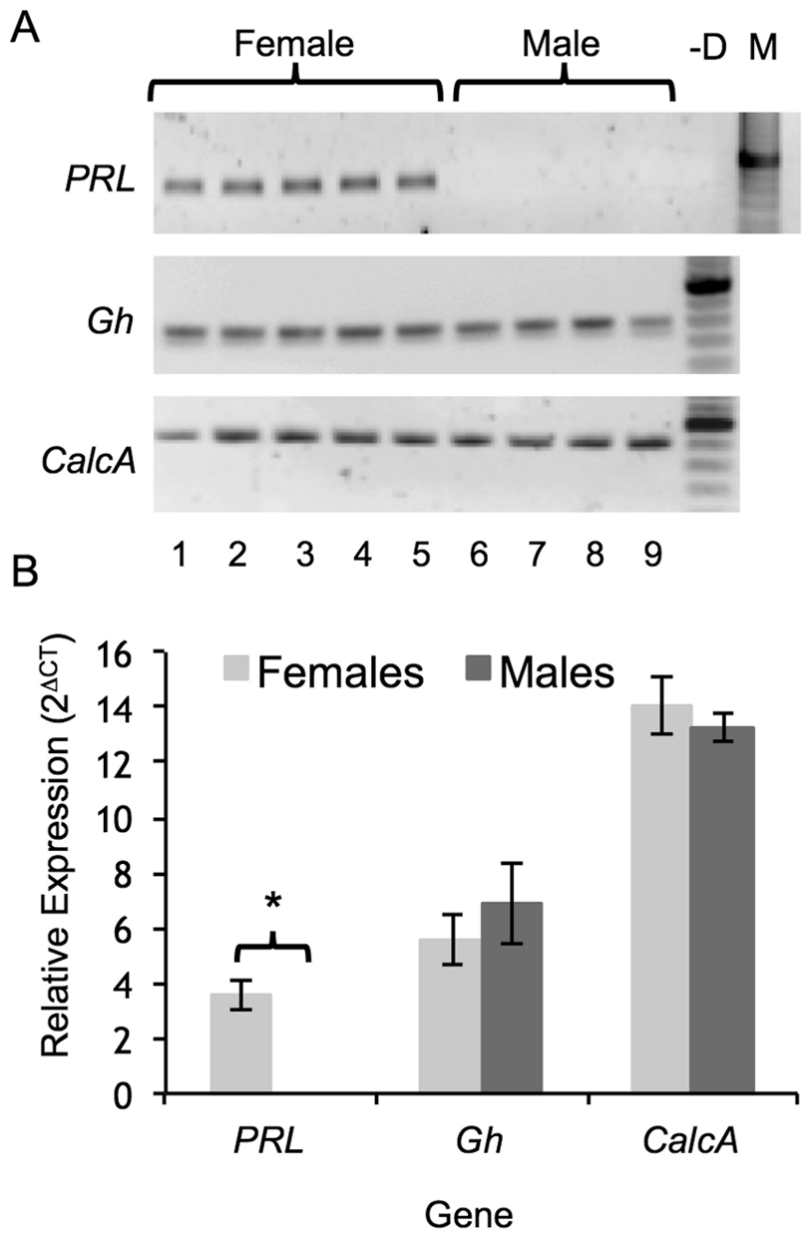
Analysis of gene expression. PCR was used to determine the expression profile of *PRL*, *Gh* and *CalcA*, which were visualised on an agarose gel (panel A). *PRL* was expressed in the female mice (n = 5) but was absent in the males (n = 4). However, *Gh* and *CalcA* were expressed in both sexes. A control consisting of no DNA template (-D) was also included. Subsequent quantitative analysis (panel B) revealed that the relative expression of *Gh* and *CalcA* were not significantly different between the sexes (p = 0.24 and p = 0.35 respectively). Error bars represent the SEM.

The expression of *PRL*, *Gh* and *CalcA* was analysed using qRT-PCR with samples of the amplicons being visualized on an agarose gel (Figure 1A). *PRL* was detected in the female mice but was absent in the male mice. However, both *Gh* and *CalcA* were detected in the cochlea of both sexes. Subsequent expression analysis revealed no significant differences in *Gh* (p = 0.24) and *CalcA* (p = 0.35) between males and females (Figure 1B).

### ABR Hearing Thresholds

**Figure 2:**
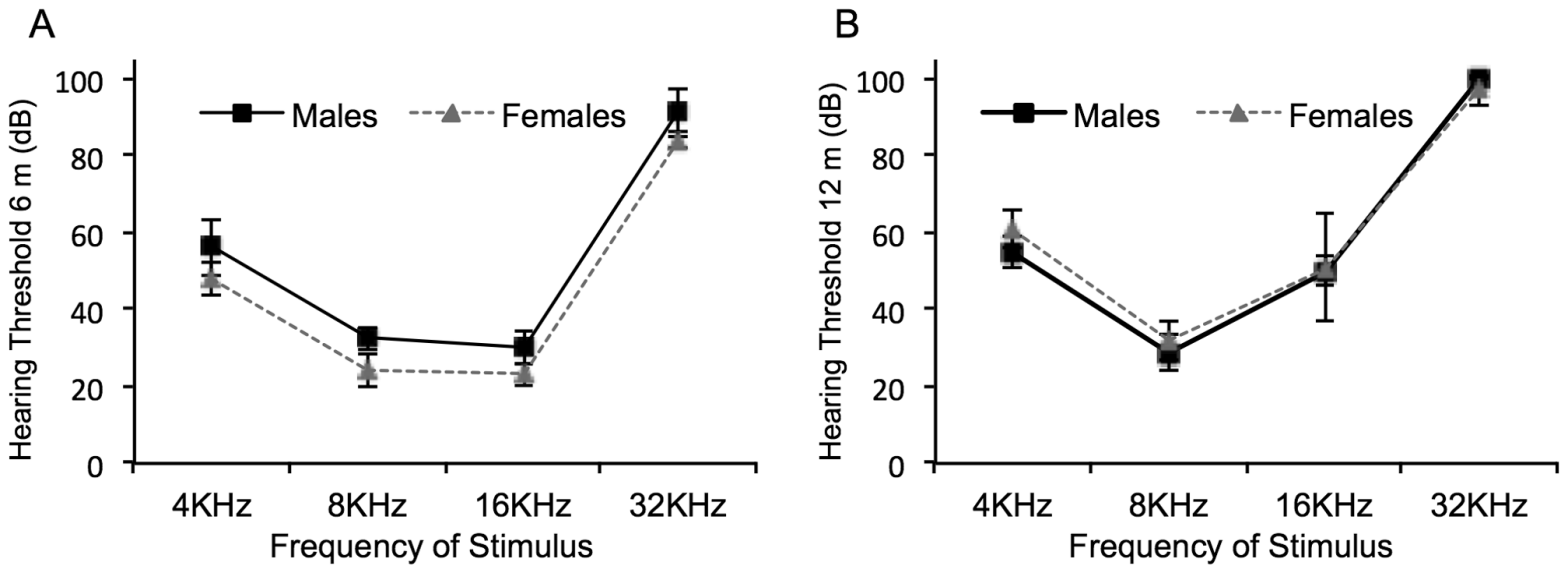
Hearing analysis using ABR. Mice were subjected to ABR analysis at 6 months of age (A) and at 12 months of age (B) to determine their hearing ability. At 6 months, female mice possessed significantly better hearing than males at 8, 16 and 32 KHz. However, at 12 months of age, the hearing of the females had worsened compared to males. Error bars equal the SEM.

**Figure 3:**
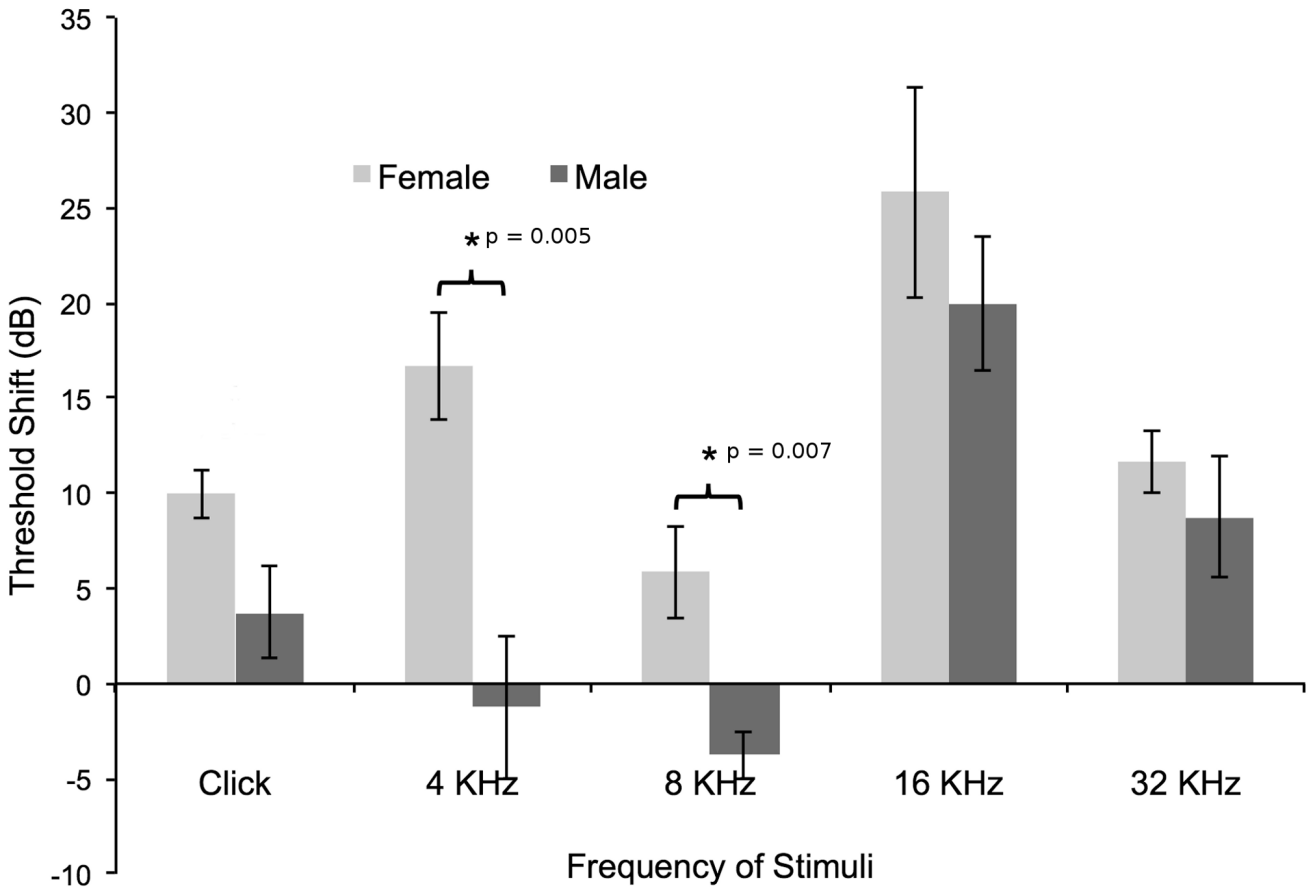
Threshold shift in hearing ability for male and female mice. The change in hearing acuity between 6 and 12 months for female and male mice were calculated for a click response and at various pure tone frequencies. It was found that females displayed a significantly greater shift in hearing threshold compared to males at 4 KHz (p = 0.005) and 8 KHz (p = 0.007). Error bars denote the SEM and * indicates a significance of p<0.05.

Mean ABR thresholds were calculated for male and female mice at 6 months and 12 months of age for a click response and at pure tones. At 6 months of age, both sexes displayed a typical ABR threshold curve (Figure 2A) for this strain of mouse, characterized by substantial hearing loss at the higher frequency of 32 KHz [18]. Additionally, although the shape of the curve was similar, males possessed significantly higher ABR thresholds than females for pure tone stimuli at 8, 16 and 32 KHz (Table 1) when analyzed using the Wilcoxon Rank Sum/Mann-Whitney U-Test. However, at 12 months of age, the situation appeared to reverse with the males recording statistically similar thresholds compared to the females for all corresponding stimuli (Figure 2B). Both sexes essentially recorded complete hearing loss at 32 KHz with thresholds above 95 dB (Table 1). Further analysis calculating the ABR threshold shift between 6 and 12 months revealed significant differences in loss of hearing between females and males (Figure 3) when analyzed using Repeated Measures ANOVA. For a click stimulus it appeared that females suffered a greater threshold shift of 10 dB ±1.29 compared to males, which demonstrated an ABR threshold shift of 3.75 dB ±2.39. However, this was not considered significant (F = 5.12; p = 0.058). For a pure tone stimulus at 4 KHz, females recorded a significantly (F = 15.89; p = 0.005) greater increase of ABR threshold of 16.66 dB ±2.79 compared to males, which recorded a reduction of 1.25 dB ±3.75. This result is also reflected in the calculations at 8 KHz, which shows females recording a mean threshold increase of 5.83 dB ±2.39, while the males recorded a threshold reduction of 3.75 dB ±1.25 (F = 14.44; p = 0.007). No significant difference was detected between females and males at 16 KHz and 32 KHz (F = 0.881; p = 0.380 and F = 1.89; p = 0.212 respectively).

**Table 1:** ABR analysis of mice at 6 and 12 months of age.

	Mean Hearing Threshold (dB) at 6 Months					Mean Hearing Threshold (dB) at 12 Months				
	Male		Female			Male		Female		
Test	Mean	SD	Mean	SD	P	Mean	SD	Mean	SD	P
Click	40	7.07	33	2.74	0.059	43.75	4.79	43	2.45	0.893
4KHz	56.25	7.5	48	4.47	0.076	55	4.08	61	4.9	0.090
8KHz	32.5	2.89	24	4.18	0.023*	28.75	4.79	32	5.1	0.373
16KHz	30	4.08	33	2.74	0.028*	50	4.08	51	13.93	0.711
32KHz	91.25	6.29	84	2.24	0.031*	100	0	97	4	0.180

* denotes a significant p value (p<0.05).

### Analysis of Bone

#### Bone Mineral Density

**Figure 4:**
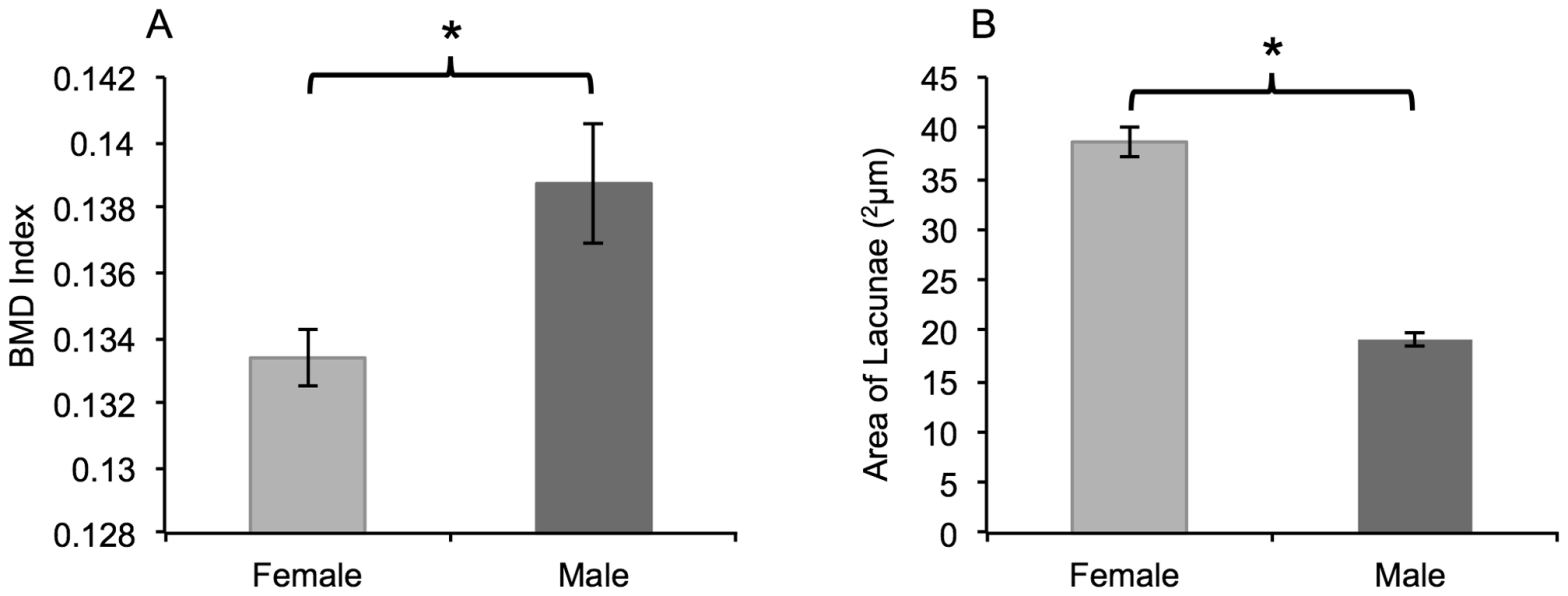
Analysis of cochlea bone. Bone mineral density of the cochlea was calculated for female and male mice using microCT scanning (panel A). Female mice possessed a significantly lower BMD index than male mice (p = 0.014). Subsequent analysis revealed that the average size of the osteocyte lacunae was significantly larger in the female mice compared to the males (p<0.001). Error bars denote the SEM and * indicates a significance of p<0.05.

Micro-CT scanning was used to calculate the BMD of cochlea of mice (Figure 4A). The BMD differed significantly (p = 0.014) between the female and male mice, which were 0.133±0.009 and 0.139±.002 respectively. This shows female mice possessed a lower BMD.

#### Area of Lacunae

The mean area of a sample of osteocyte lacunae in the cochlea of female mice was calculated to be 36.62 ^2^µm ±1.43. The mean area of a sample of osteocyte lacunae in the cochlea of male mice was calculated to be 19.12 ^2^µm ±0.69 (Figure 4B). A Students T-test revealed that the difference in mean lacunae area was significantly higher in the female mice compared to the males (p<0.001).

### OPG:RANKL Ratio

**Figure 5:**
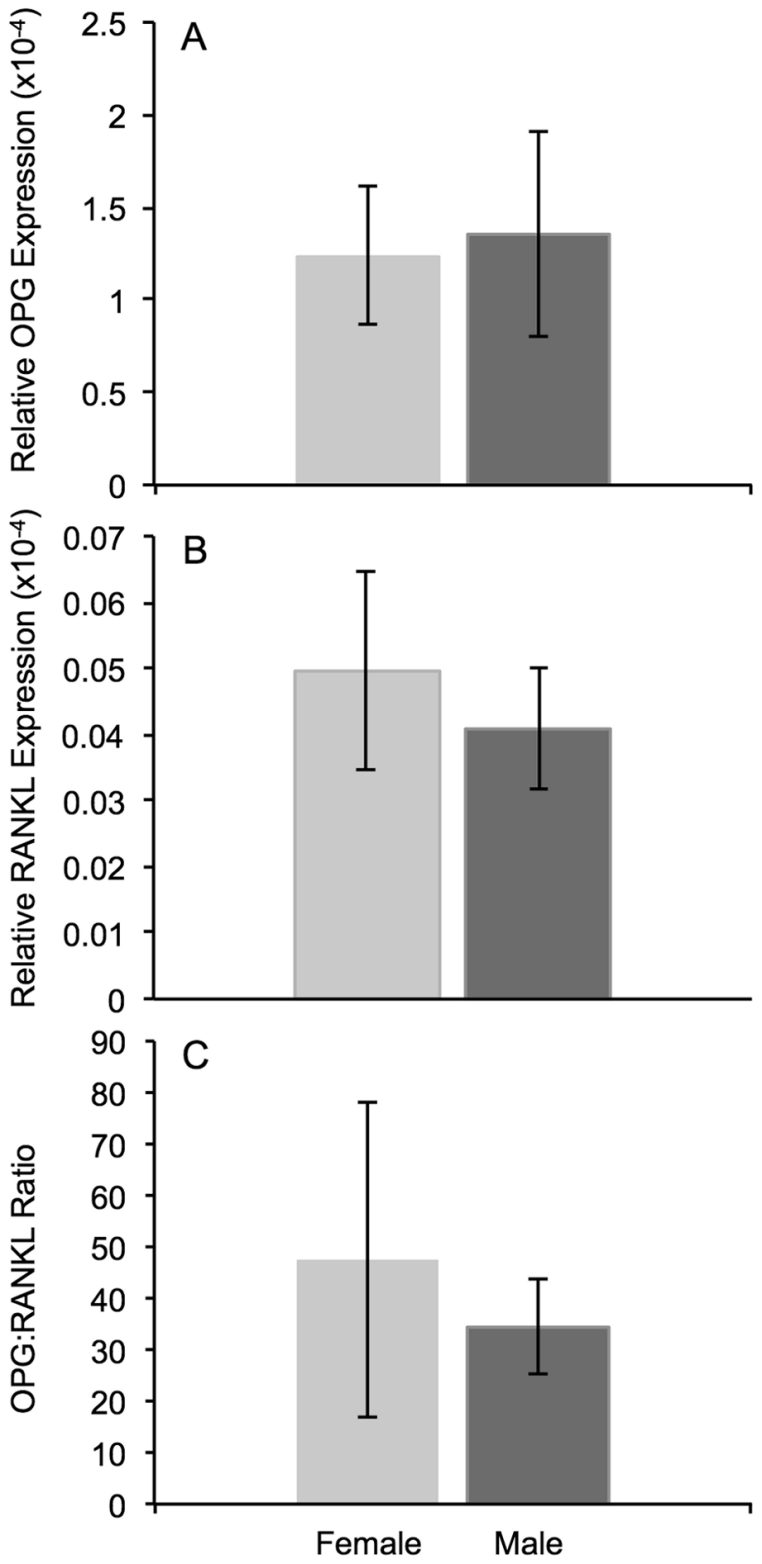
Calculation of expression levels for OPG, RANKL and their ratio. Analysis using qRT-PCR revealed that the relative expression of OPG (panel A) and RANKL (panel B) were not significantly different (p = 0.38 and p = 0.14 respectively) between females and males. Subsequent analysis of the OPG:RANKL ratio (panel 5C) also did not reveal a gender bias (p = 0.36). Error bars denote the SEM.

The relative expression of OPG and RANKL were calculated using qRT-PCR and the ratio determined for each of the sexes. A significant difference was not detected between females and males in the expression level of OPG (p = 0.38, Figure 5A) and RANKL (p = 0.14, Figure 5B). Consequently, the OPG:RANKL ratio (Figure 5C) also did not reveal a gender bias, which was 47.5∶1±30.9 for females and 34.6∶1±9.2 for males (p = 0.36).

### Histology and Staining

#### TRAcP staining for osteoclasts

Cochlea sections of both female and male mice were negative for TRAcP staining in all areas. However, positive controls stained strongly for activated osteoclasts (images not shown).

#### H&E Staining for changes in bone

**Figure 6:**
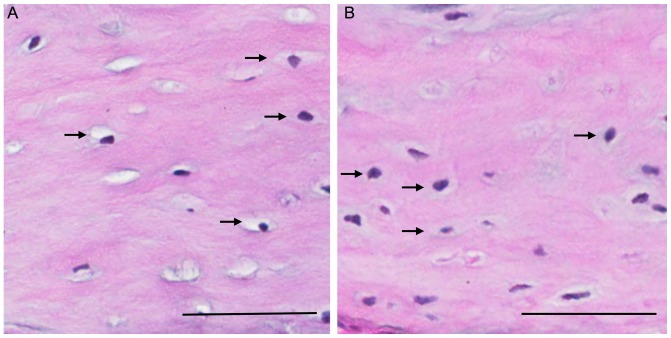
Representative histological sections of cochlea bone for female and male mice. H&E stained sections of cochlea bone revealed that the osteocyte lacunae (arrows) appeared larger in the female mice (panel A) compared to the male mice (pane B). Scale bars equal 50 µm.

Morphological observations of H&E stained sections indicated that the osteocyte lacunae appeared larger in the females compared to the males (Figure 6).

## Discussion

We have found that localised *PRL* expression in the cochlea of aged BALB/c mice demonstrated a gender bias, insomuch as it was only detectable in females. The expression of *Gh* and *CalcA* seemed to be independent of *PRL* expression. Additionally, *PRL* expression correlated to an increase in hearing thresholds at certain frequencies. Furthermore, a reduction of cochlea BMD also correlated to *PRL* expression in the female mice. However, our hypothesis that this was due to changes in the RANKL:OPG ratio could not be proven.

ARHL is a broad term used to describe an increase in hearing threshold associated with age. It is a condition that is asynchronous with the age-related condition of other organs of the body, and singularly interferes with the quality of life [19]. While ARHL is multifactorial in its pathology, there is general agreement there is a genetic component. In C57BL/6J mice, the *cdh23ahl* allele was found to be a major contributing factor for premature hearing loss [20]. However, subsequent studies in a wide variety of inbred strains have found the contribution of this gene to be highly variable and conclude the necessity of other contributing genes [21]. Currently, while there are a large number of publications describing different genes and potential mechanisms in mice, none have so far been proven as a definitive cause for ARHL in the general population. Considering the high volume of contrasting research, it may be true that there is more than one form of ARHL resulting from one of many different types of mechanisms or altered genes as postulated by others [22], [23].

We have previously shown *PRL* to be expressed in the cochlea of aged mice, and that this expression was localised to the cells of the spiral ganglion and marginal cells of the stria vascularis [15]. In addition, the expression of *PRL* seemed to correlate to a similar upregulation of growth hormone (*Gh*) and a downregulation of a known *PRL* inhibitor calcitonin (*CalcA*). Furthermore, we also demonstrated that the *PRL* receptor (*PRLR*) was located in most structures of the inner ear. Previous research by others have established a link between hyperprolactinemia and altered bone metabolism [13], [24]–[26]. In addition, hyperprolactinemia has been shown to cause bone related hearing loss [14]. In this present study we have shown that the expression of *PRL* in the cochlea of BALB/c mice seems independent of *Gh* and *CalcA* expression. In the pituitary, which is the primary site of *PRL* expression, these genes are regulated by the same mechanism [2]. However, at extrapituitary sites, *PRL* expression is predominantly controlled by elements within the superdistal promoter. This regulation is disassociated with *Gh* and *CalcA* expression, in addition to being independent of *PRL* production in the pituitary [27]. Our current results of *PRL* expression within the cochlea are is in keeping with these findings. However, *PRL* expression did correlate to gender (where it was only expressed in female mice), an increase in the ABR hearing threshold and a reduction of the BMD of the otic capsule.

These results seem to compare favorably with clinical ARHL in humans. In terms of ARHL progression, recent research has correlated the severity of hearing loss to the extent of degeneration of many inner ear structures, including the stria vascularis, ganglion cells, inner and outer hair cells [28]. Additionally, for humans, there is a gender bias in rates of hearing loss similar to that of the BALB/c mice used in our study [29]. Briefly, the research by Sharashenidze et al demonstrated that in the relatively early ages of 30–59 years, threshold elevations appear more rapid in males than in females. Thereafter, in the latter ages of 60–79 years, the threshold shifts become steeper in females. As a result, the gender differences in hearing become smoothed. However, the cause of the degeneration and the reported gender bias has yet to be determined. The results seen in our study support both of these previous findings. That is, female mice possess lower hearing thresholds than males at 6 months of age, but exhibit an accelerated loss of hearing compared to males between 6 and 12 months at a number of frequencies. Additionally, the relative abundance of *PRLR* in most areas of the cochlea indicates the potential for adverse metabolic change in the presence of hyper-expressed *PRL* as seen in other pathologies [25], [30], [31]. Furthermore, as stated earlier, we have shown that the sites of *PRL* expression in the cochlea are restricted to spiral ganglion cells and marginal cells of the stria vascularis [15]. These are the same cell types that exclusively possess estrogen receptors (ER) α and ERß respectively [32]. In addition, loss of ERß has also been linked to a hearing loss pathology [33]. The relationship between estrogen as an effector of *PRL* expression has been well documented [34]–[36]. This provides a strong indication that estrogen may play a role in the expression of cochlea *PRL,* and provides a clue as to why there is a gender bias in its expression. However, the mechanism by which *PRL* may be regulated by estrogen in this instance is yet to be determined.

As mentioned earlier, research has shown that estrogen-induced hyperprolactinemia is correlated with bone-related hearing loss in a guinea pig model [14]. However, the role reduced BMD has in the loss of hearing was one aspect of our study that was less clear. The BMD of the bony otic capsule was significantly reduced in female mice compared to males. However, the reduction was small and it is unclear if it would have a significant effect on hearing. Furthermore, the use of qRT-PCR was unable to detect any significant differences in the OPG:RANKL expression ratio, which were high for both sexes. Subsequent TRAcP staining also failed to reveal activation of osteoclasts in either female or male mice. Therefore, the possibility exists that BMD reduction is due to other mechanisms. One such mechanism may be osteocytic osteolysis, a process whereby osteocytes directly mobilise bone minerals for metabolic use (reviews by [37], [38]). The evidence for this occurring lies in the significantly larger lacunae seen in the female mice compared to the males. However, as other bones within the mice were not available, it is unknown if this process was due to *PRL* expression in the cochlea, or a function of other hormonal changes specific to female mice.

A limitation of this study was the lack of expression, BMD and histological data from mice at 6 months of age or less, that could be correlated to corresponding audiological data. However, this does not detract from the fact that *PRL* is only detectable in females and correlated to differences in bone mineral density and hearing function compared to males of the same age.

In conclusion, we have found *PRL* to be exclusively expressed in the cochlea of aged, female, BALB/c mice. This expression correlated to an increase in the hearing threshold between 6 and 12 months of age and a loss of BMD. However, we were not able to show a relationship between reduced BMD and a decrease in the OPG:RANKL ratio as hypothesized.
